# Supplementary material for: FastqPuri: high-performance preprocessing of RNA-seq data
Source: BMC Bioinformatics. 2019 May 3;20:226. doi: 10.1186/s12859-019-2799-0 (PMC6500068; doi:10.1186/s12859-019-2799-0)
Supplement: Supplementary file 2 — Archive of FastqPuri. Archive containing all files needed to install and run FastqPuri v1.0.6. Date stamp March 22, 2019. (GZ 47,819 kb) [file 12859_2019_2799_MOESM2_ESM.gz › FastqPuri-1.0.6/html/globals_s.html]

FastqPuri: File Members


FastqPuri


Here is a list of all documented file members with links to the documentation:

### - s -

- save\_Bfilter()
  : bloom.c
  , bloom.h
- save\_tree()
  : tree.h
  , tree.c
- size\_fasta()
  : fa\_read.c
  , fa\_read.h
- Split
  : str\_manip.h
- Stats\_TF
  : io\_trimFilter.h
- Stats\_TFDS
  : io\_trimFilterDS.h
- str\_isascii()
  : str\_manip.c
  , str\_manip.h
- strindex()
  : str\_manip.c
  , str\_manip.h
- strindexC()
  : str\_manip.c
- string\_seq()
  : fq\_read.h
  , fq\_read.c
- STRIP
  : defines.h
- strsplit()
  : str\_manip.c
  , str\_manip.h
- sweep\_fa()
  : fa\_read.c


---

Generated on Mon Mar 19 2018 23:42:01 for FastqPuri by  

 1.8.14
